# Alteration of the material properties of the normal supraspinatus tendon by nicotine treatment in a rat model

**DOI:** 10.3109/17453674.2010.524595

**Published:** 2010-10-08

**Authors:** Ryogo Ichinose, Hirotaka Sano, Koshi N Kishimoto, Naoya Sakamoto, Masaaki Sato, Eiji Itoi

**Affiliations:** ^1^Department of Orthopaedic Surgery, School of Medicine; ^2^Department of Bioengineering and Robotics, Graduate School of Engineering; ^3^Department of Biomedical Engineering, Graduate School of Biomedical Engineering, Tohoku University, Sendai, Japan

## Abstract

**Background and purpose:**

Several studies have shown that nicotine has a detrimental effect on the development of rotator cuff tear. However, little is known about its mechanism. We evaluated the effect of nicotine on the maximum tensile load, the maximum tensile stress, and the elastic modulus of the supraspinatus tendon in a rat model.

**Methods:**

27 rats were randomly assigned to 3 groups. Subcutaneously implanted osmotic pumps delivered two different concentrations of nicotine solution (high dose: 45 ng/mL; low dose: 22.5 ng/mL) or saline solution (controls) over a 12-week period. The level of serum cotinine, a breakdown product of nicotine, was evaluated. We performed tensile testing using the left supraspinatus tendon in each rat. The maximum load of the supraspinatus tendon was measured, and the maximum tensile stress and elastic modulus were calculated.

**Results:**

Serum cotinine levels showed controlled systemic release of nicotine. The maximum tensile load and stress were similar in the three groups. The elastic modulus was, however, higher in the nicotine groups than in the control group.

**Interpretation:**

In a rat model, ncotine caused a change in the material properties of the supraspinatus tendon. This change may predispose to a tear in the supraspinatus tendon.

Cigarette smoke is composed of a large variety of substances including nitrogen, oxygen, and carbon dioxide. Among these substances, nicotine is a highly toxic alkaloid that constitutes one of the addictive components of tobacco (Syversen et al. 1999).

The relationship between smoking and rotator cuff tear has been reported by several authors. Itoi et al. (1996) reported that smoking was a risk factor for rotator cuff tear. They clearly showed that the more the individual smoked, the greater was the tear size. More recently, Baumgarten et al. (2009) also confirmed that there was a dose-dependent and time-dependent relationship between smoking and rotator cuff tears. Kane et al. (2006) reported that macroscopic rotator cuff tears and microscopic rotator cuff degeneration were seen more frequently in cadavers of patients with a history of smoking than in those without such histories. Mallon et al. (2004) reported that smokers showed less improvement after rotator cuff repair surgery than non-smokers.

The effects of nicotine on the mechanical properties of tendon tissue are unclear. Duygulu et al. (2006) reported that nicotine impaired the Achilles tendon healing after surgical repair in a rabbit model. Galatz et al. (2006) reported that the systemic administration of nicotine delayed the tendon-to-bone healing in a rat shoulder model. In their study, the mechanical properties of the repaired tendon in the nicotine group lagged behind those in a group treated with saline. Both studies focused on the effects of nicotine on the healing process of repaired tendons. To date, no studies have been published on the effects of nicotine on the mechanical properties of normal tendon tissue.

We determined the effect of nicotine on the maximum tensile load, the maximum tensile stress, and the elastic modulus of the normal supraspinatus tendon.

## Materials and methods

### Animal models

The study was conducted under a protocol approved by the Animal Experimentation Committee of our institution (19HpA-6, 20HpA-76).

We used 12 male Sprague-Dawley rats (350–400 g). Osmotic pumps (2ML4; Durect, Cupertino, CA), which were designed to allow the continuous infusion of test drugs over a period of 4 weeks, were implanted subcutaneously along the spine. Before implantation, the pumps were filled with nicotine (148-01212; Wako Pure Chemical Industries, Osaka, Japan) at a concentration of 45 ng/mL (high-dose (H)) or 22.5 ng/mL (low-dose (L)) in saline solution for the experimental groups, and with saline solution for the control group. The concentration of nicotine in each solution was determined through pilot studies. 27 rats were randomly allocated to 3 groups (with 9 rats in each group): H, L, and controls. Only saline solution was administered in the control group.

Under general anesthesia with intraperitoneal injection of sodium pentobarbital (30 mg/kg), an osmotic pump was implanted under the skin on the back of each rat (Galatz et al. 2006). The implanted pumps were replaced twice every 4 weeks; thus, the total experimental period was 12 weeks. 1 rat in the H group died while under anesthetic for pump implant surgery and 1 rat in the L group developed skin necrosis at the site of pump insertion. Thus, 2 rats were excluded from further analysis. After completion of the 12-week treatments, all rats were killed with an overdose of sodium pentobarbital.

### Quantification of serum cotinine levels

Cotinine is one of the major breakdown products of nicotine (Wall et al. 1988). To determine serum cotinine levels, blood samples collected by cardiac puncture were centrifuged and stored at −80°C (Akhter et al. 2003). The serum cotinine levels were then measured by enzyme-linked immunosorbent assay (ELISA) using commercially available kits (T996B841, Cosmic Corporation, Tokyo, Japan).

### Mechanical testing

To assess the mechanical properties of the supraspinatus tendon, tensile testing was performed on specimens of the supraspinatus tendons from the left shoulder of each animal in the H, L, and control groups (n = 8, 8, and 9, respectively). All the tendon strips ruptured in their mid-tendon substance except for 2 specimens (1 each from the N group and the L group). These 2 tendons slipped out from the exit of tendon clump. Thus, 7 specimens from the H group, 7 from the L group, and 9 from the control group were used for assessments. Specimens of the supraspinatus tendon-humerus complex were prepared and stored at −20°C until testing. They were gradually thawed at room temperature and the supraspinatus muscle belly was removed from the intramuscular tendon. For mechanical testing, the supraspinatus tendon strip was cut using a specially designed device with 2 parallel blades. The interval between the blades was set at 0.8 mm. Next, the humeral shaft was embedded in the plastic tubes using Bismuth alloy (Sano et al. 1997). The thickness of the tendon strip was measured with an area micrometer. This contact device essentially measures the “thickness” of the tendon above a flat platen (Thomopoulos et al. 2002). The thickness of each tendon strip was measured 3 times. The average of these 3 values was used for further analysis. The cross-sectional area of the tendon strip was calculated as the average specimen thickness multiplied by the specimen width (0.8 mm).

**Figure 1.**
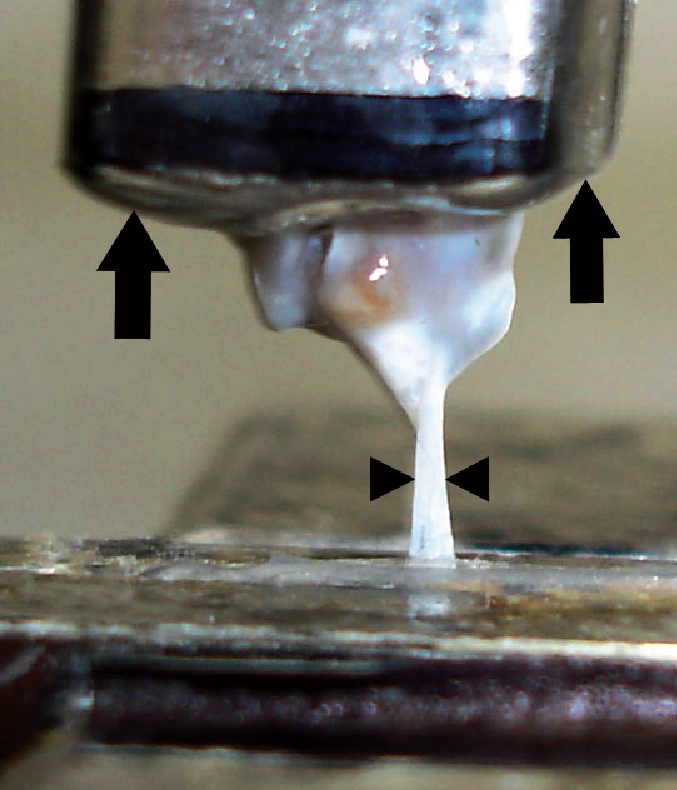
Optical strain measurement system. The humeral shaft is embedded in the metal (arrows). The supraspinatus tendon strip (arrowheads) is clamped using fine-grit sandpaper.

Tensile testing was performed with the specimen at 90 degrees of abduction (Galatz et al. 2006) using a testing machine. The block of bismuth alloy into which the humeral shaft was embedded was clamped, and the proximal end of the supraspinatus tendon strip was held with fine-grit sandpaper lined with silicone rubber to prevent slippage (Thomopoulos et al. 2002). The tendon specimen was tested along its longitudinal axis in uniaxial tension at a constant elongation rate of 3 mm/min until failure occurred. To determine tissue properties in a non-contact manner, the total specimen strain from grip to grip was measured optically with a video camera and a digital image recorder (Figure 1) (Thomopoulos et al. 2002). The load-displacement curve was obtained by this system automatically. To calculate strain, the displacement between grips was divided by the initial distance at no load. The stress was then determined using the data from the cross-sectional area. To measure the elastic properties of the tendon specimen, the elastic modulus was calculated using linear regression from the near-linear region of the stress-strain curve (Gimbel et al. 2004).

### Statistics

All data values are expressed as mean with interquartile range (IQR). Differences between groups were examined for statistical significance using the Mann-Whitney U-test. For the mechanical testing, we performed an additional statistical analysis of the 3 groups simultaneously using the Kruskal-Wallis H-test. We used the Prism 5 software package (Version 5.0a for Mac OS X). The level of statistical significance was set at p < 0.05.

## Results

### Serum cotinine levels

The mean cotinine levels for the H and L groups were 1,740 (IQR 1,383–2,080) ng/mL and 700 (556–848) ng/mL, respectively (H group vs. L group: p < 0.001; L group vs. control group: p < 0.001).

### Mechanical testing

The mean thickness of the tendon strip in the H, L, and control groups was 0.37 (IQR 0.32–0.41) mm, 0.33 (0.30–0.37) mm, and 0.38 (0.34–0.42) mm, respectively. A statistical significant difference was found between L group and control group (p = 0.03).

**Figure 2.**
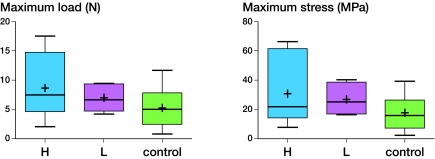
Both the maximum load (left panel) and maximum stress (right panel) in the groups given nicotine (H and L) were relatively higher than those in control group. (“+” in the box indicates the mean.)

The maximum load in the H, L, and control groups was 8.6 (IQR 4.7–15) N, 7.1 (4.8–9.3) N, and 5.3 (2.5–7.8) N, respectively (Figure 2). The maximum stress in the H, L, and control groups was 31 (IQR 14–62) MPa, 27 (17–39) MPa, and 18 (7.5–27) MPa, respectively (Figure 2). Both the maximum load and the maximum stress in the nicotine-administered groups (H and L) were higher than those in the controls, but the differences were not statistically significant.

**Figure 3.**
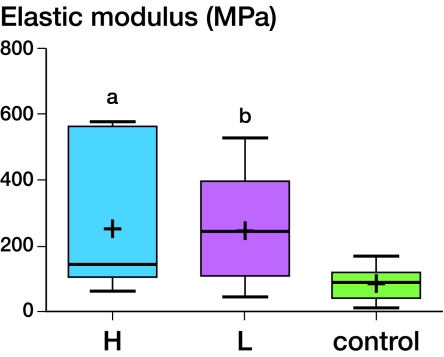
The elastic modulus of the H and L groups was higher than that of the control group (H group vs. controls: **^a^** p = 0.04; L group vs. controls: **^b^** p = 0.03).

The elastic modulus of the supraspinatus tendon in the H, L, and control groups was 252 (IQR 106–562) MPa, 249 (110–397) MPa, and 86 (43–122) MPa, respectively (Figure 3). The elastic modulus of the H and L groups was higher than that of the controls (H group vs. control group: p = 0.04; L group vs. control group: p = 0.03). In the Kruskal-Wallis H-test, the p-values for the maximum load, maximum stress, and elastic modulus were 0.3, 0.3, and 0.04, respectively. However, it should be noted that the difference in elastic modulus was judged as not being statistically significant by the post hoc test (Dunn's multiple comparisons test).

## Discussion

Cotinine is the major degradation product of nicotine metabolism and it has a long serum half-life of about 17 h, as compared to 2 h for the parent compounds, including nicotine (Benowitz et al. 1983). Measurement of nicotine has the advantage of being specific to tobacco, but it requires expensive laboratory instrumentation. Measurement of cotinine levels can provide a sensitive estimate of exposure to tobacco smoke (Jarvis et al. 1987). Because of its longer half-life, cotinine performs better in this respect than nicotine (Hill et al. 1983). Based on these advantages, we used serum cotinine levels as an indicator of systemic release of nicotine.

Contrary to our expectations, both maximum load and maximum stress were relatively higher in the groups administered nicotine than in the control group. However, none of the differences were statistically significant. On the other hand, it was interesting to note that significant differences were found in elastic modulus between the nicotine (H and L) groups and the control group; the elastic modulus was higher in the nicotine groups than in the control group.

In a previous study, stretching exercises reduced the elastic modulus of the gastrocnemius tendon (Burgess et al. 2009). Eliasson et al. (2007) found that the elastic modulus increased under conditions of disuse in a rat Achilles tendon model. In the clinical setting, it is widely believed that stretching exercises reduce the risk of tendon injury. This prevention of tendon injury may be related to decrease in the elastic modulus of the tendon. On the other hand, a tear of the supraspinatus tendon typically starts at the anterior one-third of the tendon (Tuite et al. 1998, Matsen et al. 2004), where the elastic modulus is greater than in the middle third or the posterior third (Itoi et al. 1995). Based on these findings, it is likely that the greater the modulus of elasticity, the greater the risk of tendon rupture.

Extracellular matrix is regularly synthesized or dissolved to maintain the morphology and mechanical properties of the tissue. The increased elastic modulus due to nicotine might be related to increased synthesis of collagen or reduced production of matrix metalloproteinase. It has been reported that nicotine leads to increased collagen synthesis in beagles (Ahmed et al. 1976) and that nicotine causes an increase in cross-linked collagen content, resulting in fibrosis of the myocardial interstitium in dogs (Rajiyah et al. 1996). Thus, increased collagen synthesis or increased cross-linked collagen content of fibroblasts might induce increased elastic modulus due to nicotine administration in the tendon. The nitric oxide metabolite of coronary arterial endothelium has been found to be reduced in mice exposed to cigarette smoke (Guo et al. 2006). Interestingly, the elastic modulus of the coronary artery was increased in these mice. Nitric oxide has been found to activate matrix metalloproteinase in cultured rat cells in vitro (Trachtman et al. 1996). Accordingly, inactivated matrix metalloproteinase induced by reduced nitric oxide production as a result of cigarette smoking might lead to increased elastic modulus due to nicotine administration in the tendon.

Our study has several limitations. First, our experimental period was only 12 weeks, which may have been too short to observe the changes associated with tendon degeneration. Second, the supraspinatus tendons used were normal tendons obtained from young rats. In contrast, most rotator cuff tears in human beings develop slowly over time as a result of degeneration. Thus, the animal model we used may correspond to an acute injury in young people but may not be consistent with the typical rotator cuff tears developed in the middle-aged and the elderly. Thirdly, the mean cotinine levels in our study were higher than those of smokers. The mean cotinine level in the serum of smokers who smoke more than 10 cigarettes a day is about 300 ng/mL (Jarvis et al. 1987, Wall et al. 1988). Thus, problems arise when comparing to smokers who inhale directly from cigarettes. Finally, among the many substances in tobacco smoke, we only evaluated the effect of nicotine. Tobacco ingredients other than nicotine might be responsible for the development and enlargement of rotator cuff tears in smokers.

When cutting the 0.8-mm strips and measuring the thickness of tendon specimens, there might be a risk of bias. However, the device that we used for cutting the specimen has 2 parallel blades with a 0.8-mm interval. All the specimens were cut in one procedure without changing the interval of these two blades. Thus, the width of the strips tested was always 0.8 mm. Moreover, we measured the thickness of each tendon strip 3 times using an area micrometer and the average of these 3 values was used for further analysis. We therefore believe that the possibility of technical error or measurement bias was minimal, even though these procedures were not done blind.

There was a statistically significant difference in tendon thickness between the L group and the control group. The cutting position made in the tendon strip by the custom-made device may have caused such variation in thickness, since the thickness of the specimen was not uniform in the whole tendon. However, we believe that these differences did not have any appreciable influence on the mechanical testing results since maximum stress (calculated with the thickness values) showed the same pattern as maximum load; i.e, the data in the nicotine administered H and L groups were higher than those of the control groups. Furthermore, we assume that this difference was not caused by tendon atrophy due to nicotine administration since the tendon thickness was similar in the H and control groups.

The change in material properties noted here might partly explain the adverse effect of nicotine on the supraspinatus tendon. The effect of nicotine on rotator cuff tendons may be an important factor in the clinical setting.
